# The Multidisciplinary Approach of Rectal Cancer: The Experience of “COMRE Group” Model

**DOI:** 10.3390/diagnostics12071571

**Published:** 2022-06-28

**Authors:** Stefano Scabini, Emanuele Romairone, Davide Pertile, Andrea Massobrio, Alessandra Aprile, Luca Tagliafico, Domenico Soriero, Luca Mastracci, Federica Grillo, Almalina Bacigalupo, Ciro Marrone, Maria Caterina Parodi, Marina Sartini, Maria Luisa Cristina, Roberto Murialdo, Gabriele Zoppoli, Alberto Ballestrero

**Affiliations:** 1 General and Oncologic Surgery, IRCCS Ospedale Policlinico San Martino, 16132 Genoa, Italy; davperti@gmail.com (D.P.); massobrioandrea@gmail.com (A.M.); ale.aprile89@gmail.com (A.A.); soriero.domenico@gmail.com (D.S.); 2 Department of Surgery, Ospedale Villa Scassi, 16149 Genoa, Italy; emanuele.romairone@asl3.liguria.it; 3 Department of Internal Medicine and Medical Specialties (DiMI), University of Genoa, 16132 Genoa, Italy; tagliaficoluca1992@gmail.com (L.T.); almalina.bacigalupo@hsanmartino.it (A.B.); ciro.marrone@hsanmartino.it (C.M.); mariacaterina.parodi@hsanmartino.it (M.C.P.); roberto.murialdo@hsanmartino.it (R.M.); gabriele.zoppoli@unige.it (G.Z.); aballestrero@unige.it (A.B.); 4 Pathology Unit, Department of Surgical and Diagnostic Sciences (DISC), University of Genova, 16132 Genova, Italy; luca.mastracci@unige.it (L.M.); federica.grillo@unige.it (F.G.); 5 Department of Health Sciences, University of Genova, Via Pastore 1, 16132 Genova, Italy; 6 Operating Unit Hospital Hygiene, Galliera Hospital, Mura delle Cappuccine 14, 16128 Genoa, Italy

**Keywords:** rectal cancer, laparoscopy, total mesorectal excision

## Abstract

Background: Total mesorectal excision (TME) is the gold standard to treat locally advanced rectal cancer. This monocentric retrospective study evaluates the results of laparotomic, laparoscopic and robotic surgery in “COMRE GROUP” (REctalCOMmittee). Methods: 327 selected stage I-II-III patients (pts) underwent TME between November 2005 and April 2020 for low or middle rectal cancer; 91 pts underwent open, 200 laparoscopic and 36 robotic TME. Of these, we analyzed the anthropomorphic, intraoperative, anatomopathological parameters and outcome during the follow up. Results: The length of hospital stay was significantly different between robotic TME and the other two groups (8.47 ± 3.54 days robotic vs. 11.93 ± 5.71 laparotomic, *p* < 0.001; 8.47 ± 3.54 robotic vs. 11.10 ± 7.99 laparoscopic, *p* < 0.05). The mean number of harvested nodes was higher in the laparotomic group compared to the other two groups (19 ± 9 laparotomic vs. 15 ± 8 laparoscopic, *p* < 0.001; 19 ± 9 laparotomic vs. 15 ± 7 robotic, *p* < 0.05). Median follow-up was 52 months (range: 1–169). Overall survival was significantly shorter in the open TME group compared with the laparoscopic one (Chi2 = 13.36, *p* < 0.001). Conclusions: In the experience of the “COMRE” group, laparoscopic TME for rectal cancer is a better choice than laparotomy in a multidisciplinary context. Robotic TME has a significant difference in terms of hospital stay compared to the other two groups.

## 1. Introduction

According to Global Cancer Statistics 2020 (GLOBOCAN), colorectal cancer is the second leading cause of cancer death worldwide. There are an estimated 1,900,000 new diagnoses each year, 10% of all new cancer cases.

Specifically for rectal cancer it is estimated that there are approximately 732,210 new diagnoses per year (3.8%), causing approximately 339,022 deaths per year (3.4%) [1].

The introduction of total mesorectal excision (TME), first described by Heald et al. in 1972, has improved surgical outcomes in the past few decades. Thanks to TME, now the standard practice for mid- and low-rectal adenocarcinoma [2,3], local recurrences are reduced to less than 5%. Mini-invasive resections (Laparoscopic and Robotic) for the treatment of colon adenocarcinoma are increasingly performed worldwide and show relevant benefits (shorter length of hospital stay, pain reduction, decreased intraoperative blood loss and similar oncologic results compared to open surgery), while in rectal adenocarcinoma the evidence of a better outcome after laparoscopic resection is not as robust. Several cohort studies and one small randomized controlled trial (RCT) strongly suggest that a laparoscopic approach for rectal cancer is safe [4,5,6]. Furthermore, a larger RCT (COLOR II) reports similar outcomes [5].

Laparoscopic resection for curable cancer should be part of the expertise required by surgeons who routinely operate rectal adenocarcinoma. However, the quality of laparoscopic TME in general practice may not reach the high standards reported in RCTs. Population-based studies examining outcomes after laparoscopic rectal resection are lacking, and this kind of report deserves dedicated investigations [6]. Regarding the robotic approach, it is associated with a faster postoperative recovery, but it takes longer in terms of operating time [7].

The aim of this retrospective study, performed in a high-volume center for colorectal surgery (more than 150 colorectal resections per year) was to compare outcomes after open, laparoscopic and robotic TME. The primary endpoint was the oncologic quality of surgery, the secondary ones were mortality rates at 30 days, length of hospital stay, post-operative morbidities, rates of sphincter-saving surgery, disease free survival and role of multidisciplinary dedicated staff in a tertiary level referral hospital.

## 2. Materials and Methods

Between November 2005 and April 2020, 327 consecutively diagnosed patients affected by stage I, II or III middle- or low-rectal adenocarcinoma (respectively, less than 7.5 cm and 3.5 cm from the anal verge) underwent TME at the Oncologic Surgical Unit of IRCCS San Martino IST Hospital in Genoa, Italy. These patients were selected from a total number of 658 rectal cancer resections, of which 488 were TME. All the patients were diagnosed by colonoscopy and biopsy and received a pre-operative assessment including clinical examination, complete blood tests for hematologic, liver and kidney function, CEA, CA 19.9 and whole-body CT scan. MRI and/or endorectal ultrasound (EUS) were performed in the majority of patients, unless contraindications were present.

All the patients were assessed by specialists who assessed at least 300 suspect cases of colorectal cancer per year. The MDT (multidisciplinary team) consists of surgeons, oncologists, radiotherapists, gastroenterologists and pathologists. Each of these specialists is dedicated and specialized in colorectal cancer.

New cases are discussed by our MDT on a weekly basis. Known cases are then discussed before, after surgery, after the end of adjuvant treatment and every four–six months for the first five years, then annually until death or ten years of follow-up, whichever event happens first. The patients included in the present analysis had to satisfy the following inclusion criteria: stage I, II or III disease, middle- or low-rectal adenocarcinoma, no previous pelvic surgery or radiotherapy, no conversion from laparoscopy/robotic to laparotomy (rate of conversion 5%). In our team before 2009, surgery for rectal cancer was always performed with the laparotomy technique. After 2009, we started the training course for minimally invasive surgery. Since 2012, we have acquired skills that have allowed us to indicate laparoscopic surgery for almost all patients, excluding cases in which there are absolute contraindications to pneumoperitoneum and/or serious comorbidities. Today, the selection is conducted in the same way but robotic surgery has supplanted laparoscopy for rectal surgery. All the surgeons were highly trained in colon adenocarcinoma-rectal adenocarcinoma interventions (more than 20 minimally invasive rectal resection/year each, more than 50 for two surgeons). In particular, in the last years two members of our surgical equipe were mostly dedicated to this type of surgery. Finally, all the patients had to undergo TME to be considered for the present report.

Patients with stage I disease or who rejected neoadjuvant treatment or who had contraindications to it, underwent primary surgery. Selected patients (stage II or III disease, no synchronous tumor, no contraindication to chemoradiation), received neoadjuvant chemoradiation (NCRT) (45 Gy in 25 fractions over a 5-week period with a combination of capecitabine 825 mg bid uninterrupted for 42 days). Eight to twelve (since 2009) [8] weeks after finishing the CRT, the patients underwent surgery. The type of surgery depended on the level of the tumor; one of the parameters examined was the median height of the tumor from the anal margin for the three groups considered: 3.8 ± 1.7 cm for OG, 3.5 ± 1.9 cm for LG and 2.96 ± 1.8 for RG.

We performed a TME procedure with abdominoperineal resection (APR) in case sphincter preservation was deemed unfeasible. Otherwise, low anterior resection (LAR) was performed. High ligation of the inferior mesenteric artery was not routinely practiced, in particular in dolichosigma, which gave the opportunity to perform a tension-free anastomosis. All resected specimens were examined by dedicated pathologists, according to a standardized histopathological protocol. This included: (a) a thorough evaluation of pTNM category, with a report concerning the total number of resected nodes and the number of positive nodes; (b) the examination of all lymph nodes for the presence of histologically documentable disease; (c) nodal examination continuing until at least 12 nodes could be identified. If fewer than 12 lymph nodes were found, consideration was given to placing the fatty tissue surrounding the resected viscera into a lymph node-highlighting solution [9].

Histopathological tumor regression after neoadjuvant radiochemotherapy was classified according to the Dworak score [10]. As described by Quirke and Nagtegaal [11,12], we followed a strict protocol ensuring the assessment of not only lymph nodes, but also proximal and distal resection margins, the circumferential resection margin (CRM), and the grading of integrity of the mesorectum. The pathologists, as the surgeons, were dedicated to colorectal diseases or chief consultants with long-standing experience in digestive disease assessment. The patients underwent standard follow up, which was written in literature.

### Statistical Analysis

Excel (Microsoft^®^) was employed to keep track of patient-related records. A descriptive analysis of the data was conducted. The clinical parameters of the patients were compared in relation to the three different types of intervention through ANOVA tests (in case of two or multiple groups, respectively, and on data with normal distribution) or Kruskal–Wallis test or Mann–Whitney two-sample statistic in case of non-parametric data. In case of non-continuous data the chi-squared test was utilized. Kaplan–Meier survival curves were assessed using a log-rank test to compare overall survival, whereas Cox proportional hazards regression was employed for multivariable regression analyses. A logistic analysis was conducted to calculate the odds ratio of the mortality variables in patients who underwent LG, OG and RG. The significance level was set to *p* < 0.05 for all tests. All analyses were performed using STATA^®^ SE14 (StataCorp LP, College Station, TX, USA). The analysis was adequately powered to test the equivalence of the Open Group (OG) compared to the Laparoscopic Group (LG) and Robotic Group (RG) in determining overall survival.

## 3. Results

From November 2005 to April 2020, 327 TME operations for stage I, II or III rectal cancer were enrolled. There were 201 males (61.47%) and 126 females (38.53%) with an average age of 69.24 (28–92, median age: 71) years. The OG consisted of 91 (27.83%) patients, the LG 200 (61.16%) and the RG 36 (11.01%). Demographics of the patients are reported in Table 1.

The patients were statistically different in terms of age. The three groups did not show statistically significant differences in terms of sex prevalence, site of the tumor, and type of surgery (anterior resection or abdominal perineal resection), but there was a difference in terms of ASA score (Table 1). The higher number of patients in the LG is due to our Institutional Guidelines, because since 2009 we adopted laparoscopy as the standard approach for the described condition, after an internally accepted learning curve had been reached. The three groups differed in a statistically significant manner concerning staging and number of patients undergoing neoadjuvant therapy. All the surgeons, even those executing fewer procedures in this series, performed more than 20 interventions/year (two surgeons more than 50), thus fulfilling the requirement for high skill certification, as defined by Leonard et al. [13]. The surgical, clinical and survival outcomes are described in Table 2.

The operating time was significantly different in the three groups. The number of retrieved nodes was smaller in both LG and RG compared to OG; however, this difference was no longer significant after adjusting for CRT. In fact, significantly more patients underwent neoadjuvant treatment in the LG and RG compared to the OG, but the median number of nodes was still higher than 12 (the minimum recommendation according to the UICC/AJCC criteria) [14]. There was no difference between the three groups in terms of Circumferential Radial Margin (10 mm in the OG vs. 10 mm in the LR vs. 10 mm in the RG, *p* > 0.05).

First flatus occurred after 1 day in OG and 1 day in the LG and 1 day in RG, such differences were statistically significant. The median duration of hospital stay was 11.93 days in the OG, 11.10 in the LG and 8.47 in the RG and these differences were also statistically significant.

Post-operative leakage was similar in the three groups (clinic leak evaluated only taking into account clinical signs and symptoms): the percentage of patients undergoing sphincter-saving surgery did not differ significantly in the three groups (83.5% in the OG, 74.9% in the LG and 72.2% in the RG, *p* > 0.05), while the number of ostomies performed were different in the three groups. Thirty-day mortality rate was 2.2% in the OG vs. 1.5% in the LRG (*p* > 0.05). We observed two cardiopulmonary complications in the OG group (myocardial infarction) and one in the LG group (pulmonary embolism).

**Figure 1:**
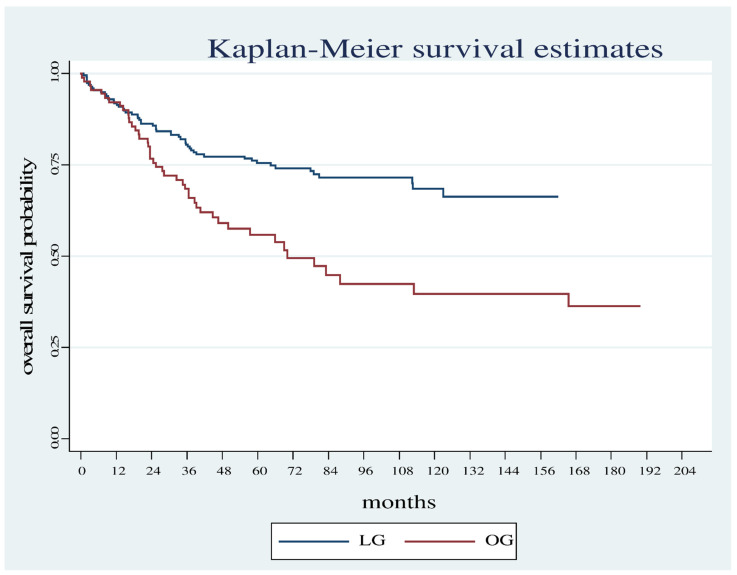
Overall survival estimates between the OG and LG.

**Figure 2:**
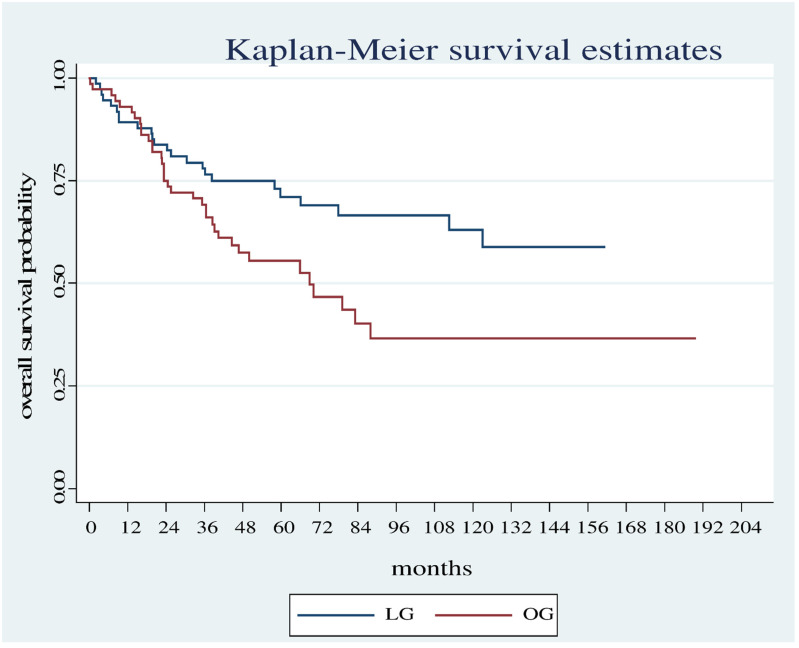
Overall survival estimates between the OG and LG in patients who did not undergo neoadjuvant therapy.

Overall survival was significantly shorter in the OG group compared with the LG group (Log Rank test: Chi2 = 13.78, *p* < 0.001) (Figure 1) even considering only the patients who did not carry out neoadjuvant therapy (Log Rank test Chi2 = 5.18, *p* < 0.0228) (Figure 2).

**Figure 3:**
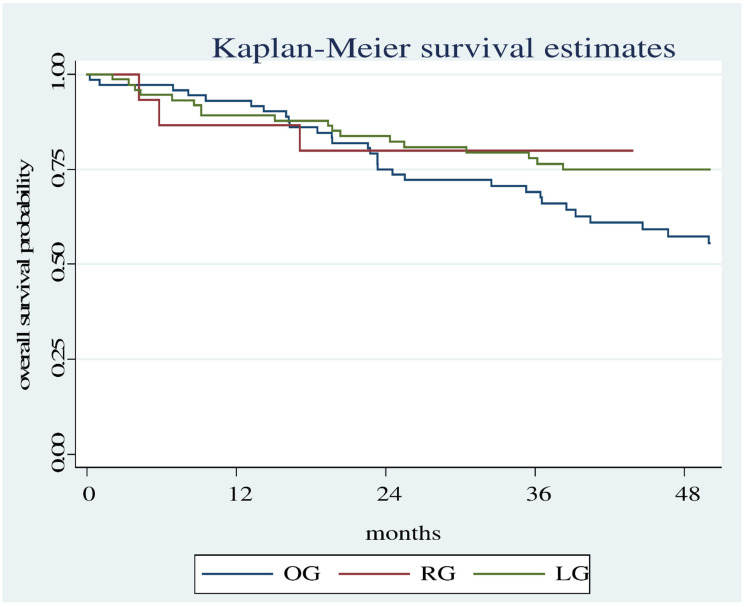
Overall survival estimates between the RG, OG and LG.

There is a statistically significant difference even when comparing all three groups (OG, LG, RG) even though the evaluation period is short (50 months). (Log Rank test: Chi2 12.23, *p* = 0.0022 (Figure 3).

Through Cox regression, we analyzed the factors that influenced mortality. Age (HR 1.06, *p* = 0.000) and stage (HR 1.34, *p* = 0.008) were found to be related to mortality (Table 3).

The logistic analysis carried out on patients who underwent intervention with LG access (Table 4) evaluating for death outcome, showed a significant increase in OR for age (OR = 1.110, *p* = 0.001), neoadjuvant therapy (OR = 5.542, *p* = 0.005) and staging (0-II vs. III) (OR = 4.556, *p* = 0.004).

On the other hand, only age was statistically significant for the patients who underwent OG (OR 1.073, *p* = 0.041) (Table 5).

Disease free survival (DFS) has also been evaluated as a function of stage to verify the presence of possible differences in terms of DFS between the three different types of access (Table 6).

## 4. Discussion

In a meta-analysis, Wang et al. [15] assessed 15 randomized clinical trials (RCTs) comparing open and laparoscopic surgery for colorectal cancer, for a total of 6,557 colorectal cancer patients. In their study, the authors concluded that laparoscopic surgery can cure colorectal cancer with no difference in terms of long-term outcomes, but with significant short-term advantages including reduced blood loss, shorter hospital stay, and faster post-operative bowel movements. However, only three RCTs from the abovementioned meta-analysis took into account rectal cancer. Other studies [3,16,17] have demonstrated that laparoscopic surgery for rectal cancer has similar safety, resection margins and completeness of resection in the short term compared to open surgery. Moreover, recovery was improved with the laparoscopic approach. Trastulli et al. [18], in his meta-analysis, observed a better outcome in the LG than in the OG regarding late intestinal adhesion-related obstructions. Only a few authors, however, such as Ng et al. [19], have so far reported long-term results. In his pooled analysis of three RCTs with a follow-up of more than 10 years, Ng confirms the long-term oncologic safety of laparoscopic surgery for rectal cancer, with no differences in local recurrence, cancer specific survival, and overall survival between the LG and the OG. Regarding the robotic approach, in the meta-analysis of Zheng et al. [7], the comparison between this type of surgery and both the open and laparoscopic approach shows a better completeness of the TME specimen in open surgery, followed by robotic and laparoscopic. There was no statistically significant difference in terms of the pathological CRM positivity, anastomotic leak, postoperative 30-day complications and mortality. In the meta-analysis of Huang et al. [20], the comparison of laparoscopic and robotic-assisted TME for rectal cancer shows a shorter operative time in the laparoscopic approach, while the robotic-assisted approach has a lower conversion rate, but there are similar pathological outcomes between these two types of surgery for rectal cancer.

In the present study, we evaluated the short- and long-term outcomes of a series of 327 consecutively treated patients undergoing TME with either a laparoscopic (the LG), robotic (the RG) or open (the OG) resection approach from 2005 to 2020. All the patients on whom we report here were operated on in the same center by surgeons with advanced training in colorectal surgery. All three groups were similar in terms of tumor site and type of operation (LAR and APR), but differed for numerosity, age, stage and indication for preoperative chemoradiation. This bias is due to the fact that, since 2009, we routinely adopted NCRT, followed by laparoscopic/robotic TME, as our internal institutional standard for stage II–III mid- and lower-rectal cancer patients, satisfying the criteria detailed in the Methods section. As a consequence, we have operated on fewer and fewer patients with an open approach over the last eleven years. The higher proportion of patients undergoing neoadjuvant CRT in the LG and RG may explain the lower stage recorded at surgery compared to the OG patients.

The primary goal of our study was to assess both short- and long-term surgical and clinical outcomes. In the short term, we did not observe differences regarding distal clearing, circumferential resection margin, and quality of specimens from TME procedures. In a randomized, two-arm, equally sized series of 340 consecutive patients assigned to receive either open or laparoscopic surgery, Jeong et al. [21] showed similar results. Likewise, Trastulli et al. [18] showed no difference in terms of proportions of interventions with a positive circumferential margin in the two groups. In our study, we observed a lower median number of lymph nodes harvested in the LG/RG vs. the OG group. However, this difference was no longer significant after adjusting for CRT, which is more often performed in the LG and RG patients. As we previously reported [22], the effect of neoadjuvant CRT is likely to affect the number of nodes harvested at surgery, even upon assessment by dedicated pathologists. Nonetheless, the median number of harvested nodes was on average higher than the minimal requirements (12 or more) suggested by common-use guidelines [23]. In respect to our findings, literature reports show some discordance, for example, Doll et al. [24] compared 102 Ut3 rectal cancer patients receiving neoadjuvant CRT with 114 Ut3 patients undergoing primary surgery followed by adjuvant CRT. After neoadjuvant CRT, both total node yield and number of tumor-positive nodes were significantly lower in the first arm, but this had no prognostic impact on overall survival. On the other hand, Schiphorst et al. [4] reported more nodes harvested in their LG, while Trastulli et al. [18] and Penninckx et al. [6] showed no difference in number of nodes harvested. Furthermore, Huang et al. [20] reported no difference in terms of nodes harvested between LG and RG. Confounding variables, such as surgeon or pathologist training, or the method for node harvesting, likely play a role in such variability, although we have to remark that it is biologically plausible to find fewer nodes, either involved or free from disease, after neoadjuvant CRT.

In our series, a shorter hospital stay was observed in the RG. These data are in accordance with the literature [25]. Larger studies have indeed also shown a shorter hospital stay with the laparoscopic approach [26,27]. Moreover, we speculate that in our patient basin (the Liguria region, which hosts the eldest population in Europe and the second eldest population in the world after Japan), fast track discharge protocols may be difficult to apply for both biological and social reasons even in the LG and RG. Nonetheless, our hospital stays constantly reduced over time.

As reported in other series [28], we showed an improvement in first bowel movement in the LG and RG groups. No significant difference was instead found in the frequencies of anastomotic leaks and in early 30-day mortality rate. In particular, we observed two cardiopulmonary complications in the OG group (myocardial infarction) and one in the LR group (pulmonary embolism), and two surgery-related deaths in the LG (sepsis in anastomotic leakage). These outcomes are in agreement with the literature, as demonstrated by the most authoritative non-inferiority studies published [16,21,29].

A fundamental finding of our analysis is that we observed different results in the two groups (OG and LG, since RG group has a shorter follow-up) concerning long-term oncologic outcomes. We believe that only a fastidious analysis of such parameters, with results clearly pointing towards the proficiency of a team of specialist surgeons, should entitle a center to pursue any given complex surgical approach. Since 2005, we have maintained a thoroughly annotated database, keeping track of and analyzing several factors. Over these 15 years (median follow-up of our patients: 40 months), neither surgical approach (open vs. laparoscopic vs. robotic), nor surgeon, pathologist, ASA score, total number of nodes harvested, or performing neoadjuvant CRT, has affected oncologic outcomes of our patients. In all groups, only CRM positivity affected oncologic results. Our findings support the concept that laparoscopic and robotic surgery for rectal cancer are two safe oncologic procedures when performed by experienced surgeons, but also highlight the relevance of highly trained pathologists [30] to avoid spurious cancer downstaging. Although the operative method of the pathologists is not blinded, we have to point out that the present manuscript is focused on clinical practice and is not a clinical trial, but rather a monocentric experience. As such, it would be unethical not to give appropriate information to our pathologists concerning the type of surgery that the patient underwent. We have, however, acknowledged a potential bias of inspecting surgical specimens with more care that come from mini-invasive procedures.

Some studies [31,32,33] showed that the laparoscopic approach is an independent predictor of better overall survival after colorectal surgery. However, more than half of the patients enrolled in those studies were affected by colon adenocarcinoma. Hence, their results may not extend to rectal adenocarcinoma. Instead, Ng et al. [19] reported that, in their experience, the laparoscopic approach was not a predictor of better survival. The authors conclude that it is not essential to demonstrate a survival benefit for laparoscopic surgery compared to the open approach to justify the role of the former procedure in the management of rectal adenocarcinoma. Indeed, Ng et al. [19] hold that the long-term oncologic safety of the laparoscopic approach for such disease can be confirmed if the oncologic outcomes and survival rates are noninferior compared with the open approach.

In our study we did not evaluate the overall survival and the disease-free survival of RG because of the short observation period. Regardless, Wilder et al. [34], in their study, reported no difference in terms of overall survival and disease-free survival between RG and LG.

Our study has several limitations. First, it is a retrospective study, with no randomization. However, it proved unfeasible to design a RCT for rectal adenocarcinoma in an institution with long-standing experience in colorectal mini-invasive surgery such as ours is. The absence of randomization is counterbalanced in our case set by an appropriate patient selection, and by the attested proficiency of the surgeons and pathologists dedicated to this type of surgical intervention. Second, NRCT was more frequently adopted in the LG and RG. As previously published [22], in our experience this may affect short-term oncologic outcomes such as the number of harvested nodes. However, as pointed out above, the median value was higher than 12, and this finding does not seem to affect long-term survival after adjusting for the type of CRT adopted (neoadjuvant vs. post-interventional).

In conclusion, our findings showed that laparoscopic resection for stage I, II, III rectal adenocarcinoma can provide comparable survival and oncologic long-term outcomes in an experienced, dedicated center. We must remark, in line with other authors [13,35,36,37], that mini-invasive resection is amply justified only when performed by qualified colorectal surgeons, with dedicated pathologists and in a multidisciplinary context. As in several disciplines where no single expert is sufficient to oversee the completion of a long-standing project, rectal adenocarcinoma management requires the intertwined expertise and the continuous mutual confrontation of professionals: only through this process can we achieve an ever-greater shared know-how and we can reach a critical competence and knowledge mass. We believe that our experience will be of further help in the process of standardizing the laparoscopic and also the robotic practice in rectal adenocarcinoma, as it was in the case of colon adenocarcinoma.

## 5. Conclusions

In selected patients treated by skilled surgeons, the laparoscopic approach provides comparable long-term oncologic outcomes to the open surgery. We could not evaluate the long-term outcomes for the robotic approach because of the short follow-up, but our data show better results compared to the other two approaches in terms of hospital stay. However, differences in patient allocation could introduce bias in our analyses, as such we caution the reader against overinterpreting the presented results.

## Figures and Tables

**Table 1:** Demographic characteristics of the patients enrolled in the present study.

PARAMETERS	Levels or Values	Open Group (N. 91)	Laparoscopic Group (N. 200)	Robotic Group (N. 36)	Total (N. 327)	*p*-Value
Age (years)	median (IQR)	76 (68–82)	68.5 (60–76)	72 (58–79)	71 (62–78)	*p* = 0.0001
Sex	MALE	50 (54.95)	125 (62.50)	26 (72.22)	201 (61.47)	NS
(N, percent)	FEMALE	41 (45.05)	75 (37.50)	10 (27.78)	126 (38.53)	
BMI	median (IQR)	24.49 (23.15–26.23)	24.22 (23.14–26.12)	24.80 (24.09–26.17)	24.44 (23.38–26.12)	NS
ASA score (N, percent)	I II III IV	20 (21.98) 39 (42.86) 28 (30.77) 4 (4.4)	54 (27.00) 105 (52.50) 39 (19.50) 2 (1.00)	4 (11.11) 22 (61.11) 10 (27.78) 0 (0.0)	78 (23.85) 166 (50.76) 77 (23.55) 6 (1.83)	*p* = 0.036
Tumor site	MID	53 (41.76)	91(45.50)	14 (38.89)	158 (48.32)	NS
(N, percent)	LOWER	38 (58.24)	109 (54.50)	22 (61.11)	169 (51.68)	
Tumor grade (N, percent)	G1 G2 G3 Gx	2 (2.20) 64 (70.33) 17 (18.68) 8 (8.79)	7 (3.50) 86 (43.00) 11 (5.50) 96 (48.00)	0 (0.00) 14 (38.88) 2 (5.56) 20 (55.56)	9 (2.75) 164 (50.15) 30 (9.18) 124 (37.92)	*p* < 0.001
Stage (N, percent)	0 I II III X	0 (0.00) 14 (15.38) 38 (41.76) 38 (41.76) 1 (1.10)	20 (10.00) 57 (28.50) 56 (28.00) 52 (26.00) 15 (7.50)	5 (13.89) 10 (27.78) 12 (33.33) 8 (22.22) 1 (2.78)	25 (7.65) 81 (24.77) 106 (32.42) 98 (29.97) 17 (5.20)	*p* < 0.001
Type of surgery (N, percent)	LAR APR	76 (83.52) 15 (16.48)	148 (74.00) 52 (26.00)	26 (72.22) 10 (27.78)	250 (76.45) 77 (23.55)	NS
NCRT (N, percent)	NO YES	73 (80.22) 18 (19.78)	76 (38.00) 124 (62.00)	15 (41.67) 21 (58.33)	164 (50.15) 163 (49.85)	*p* < 0.001
DFS (months)	Median (IQR)	40 (18–79)	69 (35–104)	30 (26–40)	53 (25–94)	*p* = 0.0001

Legend: O: open group; L: laparoscopic Group; R: robotic group; vs.: versus; LAR: low anterior resection; APR: abdominoperineal resection; NCRT: neoadjuvant chemoradiation; DFS: disease-free survival.

**Table 2:** Surgical, clinical and survival outcomes of the described case set.

PARAMETERS	Levels or Values	Open Group	Laparoscopic Group	Robotic Group	*p*-Value
Operating time (minutes)	median (IQR)	150 (105–190)	180 (150–222)	225 (210–252)	*p* < 0.001
Nodes (n)	median (IQR)	18 (12–23)	14 (9–19.5)	14.5 (11–17.5)	*p* < 0.001
Distal resection margin (mm)	median (IQR)	3 (2–4)	3 (1.7–4.5)	2 (1.1–3.95)	NS
CRM length (mm)	median (IQR)	10 (2.25–15)	10 (5–15)	10 (5–15)	NS
Complete TME N° (percent)	YES	76 (83.52)	177 (88.50)	33 (91.67)	NS
	INCOMPL	7 (7.69)	9 (4.50)	0 (0.00)	
	NEARLY COMPLETE	5(5.49)	11 (5.50)	3 (8.30)	
	NV	3 (3.30)	3 (1.50)	0 (0.00)	
Ostomy N° (percent)	DEF	34 (37.36)	54 (27.00)	9 (25.00)	*p* = 0.020
	TEMPORARY	45 (49.45)	128 (64.00)	27 (75.00)	
	NO	12 (13.19)	18 (9.00)	0 (0.00)	
Hospital stay (days)	median (IQR)	11 (8–14)	8 (7–13)	8 (6–10)	*p* < 0.001
Flatus time (days)	median (IQR)	1 (1–2)	1 (1–2)	1 (1–1)	*p* = 0.002
Clinic leak	n° (percent)	3 (3.30)	5 (2.50)	0 (0.00)	NS
Sub-clinic leak	n° (percent)	4 (4.40)	11 (5.50)	0 (0.00)	NS
Intrahospital mortality	n° (percent)	2 (2.20)	3 (1.50)	0 (0.00)	NS
Local recurrence	n° (percent)	6 (6.85)	10 (5.20)	0 (0.00)	NS
Metacr MTS	n° (percent)	17 (18.68)	28 (14.00)	0 (0.00)	O vs. L NS O vs. R *p* < 0.01 L vs. R *p* < 0.05
FU (months)	Median (IQR)	43 (24–78)	71 (27–104)	30 (26–40)	*p* = 0.0001
180 mth OS	*%*	50.55	29.00	3.70	O vs. L *p* < 0.001

Legend: O: open group; L: laparoscopic Group; R: robotic group; vs.: versus; CRM: Circumferential Radial Margin; TME: Total mesorectal excision; MTS: Metastases; FU: failure units.

**Table 3:** Cox regression.

Covariates	Hazard Ratio	Standard Error	Z	*p*-Value	95% CI	
					Lower	Upper
Age (1 year)	1.058	0.014	4.35	<0.001	1.032	1.086
Sex (F vs. M)	1.112	0.230	0.51	0.609	0.741	1.668
BMI (1 point)	0.968	0.029	−1.09	0.276	0.913	1.026
Approach (OG vs. LG)	1.403	0.327	1.45	0.146	0.889	2.214
Neoadjuvant (no vs. yes)	1.210	0.304	0.76	0.448	0.739	1.981
ASA (1 point)	1.209	0.171	1.34	0.180	0.916	1.596
Stage (from stage 0 to stage X)	1.343	0.148	2.67	0.008	1.082	1.668

**Table 4:** Logistic regression LG.

Covariates	Odds Ratio	Standard Error	Z	*p*-Value	95% CI	
					Lower	Upper
Age (1 year)	1.110	0.036	3.23	0.001	1.042	1.182
Sex (F vs. M)	1.603	0.838	0.90	0.366	0.576	4.466
Neoadjuvant (no vs. yes)	5.542	3.372	2.81	0.005	1.682	18.261
BMI (1 point)	0.977	0.065	−0.35	0.728	0.856	1.113
ASA (1–2 vs. 3–4)	1.314	0.699	0.51	0.608	0.463	3.729
Grading (G1–2 vs. G3)	1.248	0.966	0.29	0.775	0.274	5.689
Stage (0–1–2 vs. 3)	4.556	2.431	2.84	0.004	1.601	12.964

**Table 5:** Logistic regression OG.

Covariates	Odds Ratio	Standard Error	Z	*p*-Value	95% CI	
					Lower	Upper
Age (1 year)	1.073	0.370	2.04	0.041	1.003	1.148
Sex (F vs. M)	1.336	0.652	0.59	0.553	0.513	3.477
Neoadjuvant (no vs. yes)	2.629	2.086	1.22	0.223	0.555	12.451
BMI (1 point)	0.929	0.055	−1.23	0.218	0.826	1.044
ASA (1–2 vs. 3–4)	1.105	0.613	0.18	0.858	0.372	3.278
Grading (G1–2 vs. G3)	1.750	1.063	0.92	0.357	0.532	5.758
Stage (0–1–2 vs. 3)	1.601	0.0791	0.95	0.341	0.608	4.217

**Table 6:** DFS as a function of the three different approaches and of the tumor stage.

	DFS (Days) Median (IQR)	*p*
STAGE 0		*p* = 0.009
OG	-	
RG	1005 (880–1062)	
LG	2164 (1658–3485)	
STAGE I		*p* = 0.0001
OG	1344 (480–1918)	
RG	1105 (817–1307)	
LG	2517 (1711–3579)	
STAGE II		
OG	1096 (573–3624)	
RG	953 (722–1198)	NS
LG	1844 (624–2727)	
STAGE III		
OG	966 (488–1867)	
RG	797 (418–951)	NS
LG	1304 (329–2402)	
STAGE X		
OG	5199	
RG	198	NS
LG	3553 (2050–4192)	

## Data Availability

The data presented in this study are available on motivated request from the corresponding author.
